# Epoetin administrated after cardiac surgery: effects on renal function and inflammation in a randomized controlled study

**DOI:** 10.1186/1471-2369-13-132

**Published:** 2012-10-03

**Authors:** Sophie de Seigneux, Belen Ponte, Lucien Weiss, Jérôme Pugin, Jacques André Romand, Pierre-Yves Martin, Patrick Saudan

**Affiliations:** 1 Service of Nephrology, Department of Medical Specialties, Geneva University Hospitals, Rue Gabrielle-Perret Gentil 4, 1211, Geneva, Switzerland; 2 Department of internal medicine, Geneva University Hospitals, Geneva, Switzerland; 3 Service of General Intensive Care, Department of Anesthesiology, Geneva University Hospitals, Geneva, Switzerland

**Keywords:** Erythropoetin, NGAL, Cytokines, AKI

## Abstract

**Background:**

Experimentally, erythropoietin (EPO) has nephroprotective as well as immunomodulatory properties when administered after ischemic renal injury. We tested the hypothesis that different doses of recombinant human EPO administered to patients after cardiac surgery would minimize kidney lesions and the systemic inflammatory response, thereby decreasing acute kidney injury (AKI) incidence.

**Methods:**

In this double-blinded randomized control study, 80 patients admitted to the ICU post-cardiac surgery were randomized by computer to receive intravenously isotonic saline (n = 40) versus α-Epoetin (n = 40): either 40000 IU (n = 20) or 20000 IU (n = 20). The study lasted one year. The primary outcome was the change in urinary NGAL concentration from baseline and 48 h after EPO injection. Creatinine, cystatine C and urinary NGAL levels were measured on the day of randomization and 2–4 days after EPO injection. To assess acute inflammatory response, serum cytokines (IL6 and IL8) were measured at randomization and four days after r-HuEPO injection. Patients and care-takers were blinded for the assignment.

**Results:**

No patient was excluded after randomization. Patient groups did not differ in terms of age, gender, comorbidities and renal function at randomization. The rate of AKI assessed by AKIN criteria was 22.5% in our population. EPO treatment did not significantly modify the difference in uNGAl between 48 hours and randomization compared to placebo [2.5 ng/ml (−17.3; 22.5) vs 0.7 ng/ml (−31.77; 25.15), p = 0.77] and the incidence of AKI was similar. Inflammatory cytokines levels were not influenced by EPO treatment. Mortality and hospital stays were similar between the groups and no adverse event was recorded.

**Conclusion:**

In this randomized-controlled trial, α-Epoetin administrated after cardiac surgery, although safe, demonstrated neither nephroprotective nor anti-inflammatory properties.

**Trial registration number:**

NCT00676234

## Background

Erythropoietin (EPO) is a hematopoietic factor mainly produced by interstitial fibroblasts in the renal cortex and outer medulla. In addition to regulating red blood cell production, this glycoprotein has pleiotropic properties. Amongst these, cytoprotection has been demonstrated in animal brain, kidney and liver [1,2]. These effects appear to be mediated by the presence of receptors that may respond to higher doses of EPO and alter cell survival, proliferation and inflammation [2,3].

Acute kidney injury (AKI) is a frequent complication in the intensive care unit. Despite decades of research for a curative treatment, therapy is essentially restricted to supportive care and mortality remains persistently high [4]. In experimental models of ischemia-reperfusion, recombinant human EPO (r-HuEPO) injection before or during the injury appears to protect against acute kidney injury [5-7]. Even delayed administration of r-HuEPO, up to 6 hours after a renal ischemic injury, seems to be nephroprotective in rats [3]. The few data available in humans do not seem to reproduce this protective effect [8].

Diagnosis of AKI in the intensive care setting is currently performed using RIFLE or AKIN criteria [9]. Mortality increases with the AKI stages using these criteria [10]. In patients undergoing cardiothoracic surgery, AKI is a relevant complication. However, its incidence is very variable (between 1 to 30%) according to definitions and patient’s profile but more frequent in valvular versus CABG [11] surgeries. Even small increases in creatinine levels are associated with substantial decrease in 30-day survival [12]. As both creatinine levels and quantification of diuresis raise some concern in the ICU setting, novel biomarkers such as Neutrophil Gelatinase Associated Lipocalin (NGAL) are studied to identify patients at risk of developing AKI earlier. The predictive value of these markers for mortality may also be higher than creatinine, even at the time of intensive care admission or postoperatively [13,14]. Urinary NGAL (uNGAL) seems to be more sensitive than plasmatic dosage. Although, no consensus definition of AKI exists using these biomarkers [15]**,** NGAL detected patients with subclinical AKI and predicted their mortality despite unchanged serum creatinine [14]. Additionally, delayed diagnosis of AKI based on change in creatinine could explain some negative interventional trials and the absence of effective therapy in AKI treatment [16].

An inflammatory response is present in most ICU patients, particularly if they undergo reperfusion injury. IL6 levels are associated with AKI in postoperative cases [17,18]. In experimental heart ischemia reperfusion and in brain ischemia, EPO treatment decreases the systemic inflammatory response as demonstrated by decreased levels of IL6, IL8 and TNF-α [19,20].

In this study, we tested the effect of two different doses of r-HuEPO on renal function administered to patients early after cardiac surgery. We used uNGAL as a surrogate of renal function as it has been described to be an earlier and more sensitive marker of AKI than creatinine. We expected to observe a deeper decrease in uNGAL levels in the EPO treatment group, which should be associated with decreasing mortality and shorter hospital stay. Furthermore, we investigated the immunomodulatory properties of EPO after cardiac surgery.

## Methods

The study took place in the intensive care unit (ICU) at the University Hospital in Geneva (Switzerland) in 2008–2009. Initially, all adults (>18 years old) admitted to the ICU and at risk for acute kidney injury (mechanical ventilation, sepsis, post-operative state, hemodynamic impairment, previous chronic kidney disease) were screened for inclusion. Patients were excluded from the study in case of: malignant hypertension or systolic blood pressure ≥150 mmHg at enrollment, Hb levels ≥120 g/l at randomization, acute coronary syndrome, pregnancy or urinary output <600 ml/12 h.

Additionally, according to the request of the national drug control organ (Swiss Medic), only patients able to read, ask questions before inclusion and sign a consent form were eligible for inclusion. Patients unable to understand the information for any reason or in an emergency situation that prevented prior consent had to be excluded. Deferred consent or informed consent obtained from health care proxy could not be used. For this reason, although all patients admitted in the ICU were first supposedly eligible for enrollment, finally only patients having “elective” cardiac surgery could be asked for consent prior to randomization and were included in this study. During surgery, patients were managed according to a published protocol [21]. The study was approved by the ethical committee from Geneva University Hospital and registered at ClinicalTrial.gov (NCT00676234).

Although this was a pilot study, we calculated that 35 patients treated by EPO and 35 controls were necessary to obtain a 95% power with α <0.05, assuming a between group difference of 45 ng/ml in our primary endpoint. Predicting a drop out of 10 patients (5 in each group), we decided to include 80 patients: 40 would be treated by EPO and 40 by placebo.

### Treatment

In this pilot study we additionally tested 2 different dosages of EPO in order to estimate a dose-dependent effect. Patients were randomly allocated to 2 treatment groups or a control group after cardiac surgery. Group 1 received α-Epoetin: 20’000ui, group 2 α-Epoetin: 40’000ui and group 3 (control group) isotonic sodium chloride. All treatments were administered intravenously as a single dose. EPO was diluted in isotonic sodium chloride. A randomization code was generated by a computer 1:1:2 and envelopes with allocation were prepared by the quality of care unit. A nurse from the Nephrology Unit opened the envelopes and prepared the syringes for injection. Investigators and patients were blinded to the treatment. Randomized injection occurred on arrival to the intensive care unit between 1 and 4 hours after surgery.

### Outcome

The primary outcome was the change in urinary NGAL concentration from baseline and 48 h after r-HuEPO injection. The secondary outcomes were changes in traditional renal function markers (serum creatinine and cystatine C) and in cytokines levels.

### Collected variables

All patients had baseline renal function (creatinine) measured at least 24 hours before the surgery. Chronic kidney disease (CKD) was defined as a GFR <60 ml/min/1.73 m^2^ using the MDRD equation, or known kidney disease. Data on comorbidities were collected including hypertension, diabetes and history of ischemic heart disease. ICU and hospital stay in days were recorded as well as mortality and initiation of renal replacement therapy. Patients were followed for 28 days, looking for possible complications related to r-HuEPO injection such as central or peripheral thrombosis or death. AKI was detected from ICU admission to the following week using serum creatinine criteria (AKIN classification). On ICU admission, blood and urine samples were taken prior to injection (= randomization time). The same samples were taken in the morning 48 h and 96 h after the injection. Serum creatinine and cystatine C were immediately measured at 3 intervals (randomization, 48 h and 96 h). Urine was collected and kept at −20° to measure NGAL at randomization and 48 h. Serum was also kept at −20° to measure cytokines at randomization and 96 h. Creatinine was measured using the Jaffe technique and Cystatine C by nephelometry. Rapid NGAL ELISA kit (Bioport Diagnostics-Gentofte, Denmark) was used to measure urinary NGAL (uNGAL) [22]. Cytometric bead array human inflammatory cytokines kit (BD Biosciences – San Jose, California) was used to measure IL-6 and IL-8. All samples for NGAL and cytokines measures were processed at the same time.

### Statistics

Continuous variables were described by mean and standard deviation when distribution was normal. When not normally distributed, continuous variables were described by medians and interquartile ranges (25th-75th). When distribution changed at different times (randomization, 48 h, 96 h), results were reported as median and interquartile range to homogenize descriptions. Numbers and percentages described categorical variables. ANOVA or Kruskall-Wallis test was used to compare continuous variables between the three randomized groups and chi-square was used for categorical variables. Paired test (parametric and non parametric) were performed to assess changes in creatinine, cystatine C, uNGAl and cytokines at different intervals (randomization, 48 h, 96 h). Post-hoc analysis and Bonferroni correction were used when appropriate. The interleukins levels were transformed to logarithmic variables for statistics comparisons in order to achieve a normal distribution.

SPSS17-0 software package (SPSS Inc., Chicago IL) was used for the analysis.

## Results

**Figure 1:**
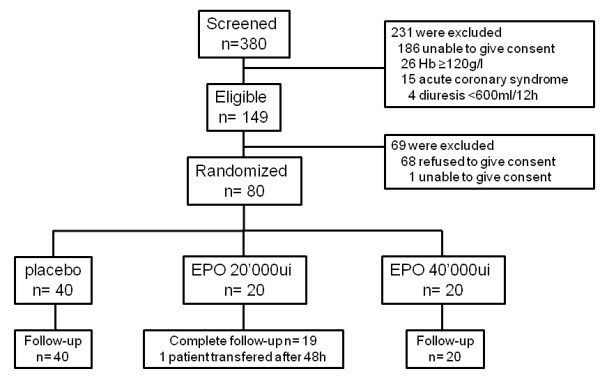
Study flow diagram.

Over 1 year, 386 patients admitted to ICU were screened for the study but only 149 met the inclusion criteria. Non eligibility was mainly due to inability to give informed consent and Hb ≥ 120 g/l. From the 149 eligible patients, only 80 patients having cardiac surgery, signed consent forms and were randomized after the surgery at ICU admission to placebo (n = 40) or EPO groups (N = 20 in each treatment group). A flow diagram of the study is depicted in Figure 1. Only 1 patient was transferred in another hospital at 48 h. Although we could not have the complete biological follow-up, we could have the information that he did not develop AKI and was alive.

Patient’s characteristics and peri-operative interventions are shown in Table 1. Results are described separately for each EPO treatment (20’000 ui *vs* 40’000ui) in order to better analyze an EPO dose-effect. The mean age was 66.3 ± 14.5 years. There were more men than women. Most patients had hypertension (68.8%), some had diabetes (28.8%) or chronic kidney disease (13.8%). Although many had a history of ischemic heart disease (41.3%), the majority of surgeries were for valvular replacement (61.5%). Only 21.6% of patients had coronary bypass surgery and 10.8% had at least 2 different interventions. There were no significant differences between randomization groups with regards to age, sex, diagnosis of chronic kidney disease, diabetes, ischemic heart disease or surgery. Baseline renal function, as assessed by creatinine levels before surgery, was also similar with a mean value of 87.8 ± 28.9 μmol/l. We reported some peri-operative characteristics such as cardiopulmonary bypass length, inotropic or vasopressor use, necessity of transfusions and emergency of the intervention that were all similar between the groups. Some patients in each group received contrast media before surgery (n = 7, p > 0.05), but none received nephrotoxic agents such as aminoglycosides or contrast media after surgery.

**Table 1:** Population and peri-operative intervention characteristics according to treatment group

Variables *	**All**	**Control**	**EPO 20’000ui**	**EPO 40’000ui**	**P∥**
	**N = 80**	**N = 40**	**N = 20**	**N = 20**	
**Population characteristics:**					
**Age in years**	66.3 ± 14.5	64.7 ± 14.7	68.9 ± 12.0	66.5 ± 16.5	0.56
**Sex**					
** Male**	56 (70)	27 (67.5)	16 (80)	13 (65)	0.52
** Female**	24 (30)	13 (32.5)	4 (20)	7 (35)	
**Hypertension**	55 (68.8)	26 (65)	17 (85)	12 (60)	0.18
**Diabetes**	23 (28.8)	14 (35)	5 (25)	4 (20)	0.44
**Chronic kidney disease**	11 (13.8)	6 (15)	2 (10)	3 (15)	0.85
**Ischemic heart disease**	33 (41.3)	17 (42.5)	9 (45)	7 (35)	0.79
**Creatinine*baseline*μmol/l**	86.4 ± 25.5	84.7 ± 26.4	92.5 ± 26.1	86.7 ± 24.0	0.79
**Intraoperative variables:**					
**Cardiopulmonary bypass min**	100 (80–136)	100 (77–120)	96 (77.5-144)	110 (83–143)	0.58
**Transfusions administred○**	28 (40)	17 (50)	6 (33.3)	5 (27.8)	0.24
**Inotropic agent use**	16 (20.3)	6 (15)	4 (21.1)	6 (30)	0.39
**Vasopressor agent use**	18 (22.8)	9 (22.5)	4 (21.1)	5 (25)	0.96
**Urgency of operation**	19 (23.8)	7 (17.5)	5(25)	7 (35)	0.32

*Age and creatinine are expressed as mean ± standard deviation and length of cardiopulmonary bypass as median (25^th^-75^th^percentiles).

Categorical variables are written as numbers of participants and percentages (%).

∥ p compares the 3 treatment groups.

○ information missing for 10 patients, 6 in the control group:% calculated for a total of 70 patients.

### Outcomes, AKI and renal function

There were no differences in hemoglobin, creatinine, cystatine C and urinary NGAL levels between the groups at randomization (Table 2).

**Table 2:** Patients’ evolution during follow-up: hemoglobin, renal biomarkers and clinical outcomes

**Variables**	**Control**	**EPO 20’000ui**	**EPO 40’000ui**	P*
**Median (25**^**th**^**-75**^**th**^**percentiles)**	**N = 40**	**N = 20**	**N = 20**	
**Hemoglobin R g/l**	97.5 (87.5-105.8)	100.5 (92.3-107)	98 (85–107)	0.41
**Hemoglobin last g/l**	100.5 (96.3-113.8)	100.0 (93–109)	101.5 (90.8-114.8)	0.69
**Renal biomarkers**				
**Creatinine R μmol/l**	78 (61–98)	94 (74.5-102)	91.5 (67.8-114.3)	0.29
**Creatinine 48 h μmol/l**	79 (65.8-88.8)	78 (62–98)	82.5 (66–114.5)	0.23
**Creatinine 96 h μmol/l**	77.5 (63.3-85.8)	75 (65–92)	87 (72.8-100.8)	0.53
**Cystatine C Rmg/l**	1.1 (0.9-1.4)	1.2 (1.1-1.5)	1.2 (1.0-1.4)	0.60
**Cystatine C 48 h - mg/l**	1.2 (1.1-1.4)	1.3 (1.1-1.6)	1.3 (1.1-1.6)	0.25
**Cystatine C 96 h mg/l**	1.3 (1.1-1.4)	1.2 (1.1-1.5)	1.3 (1.1-1.7)	0.80
**Urinary Ngal R ng/ml**	21 (5.7-44.3)	43.3 (18.3-62.9)	11 (0–76)	0.18
**Urinary Ngal 48 h ng/ml**	26.8 (12–46.7)	15.7 (2.6-39)	26 (6.2-58.8)	0.47
**Clinical outcomes**				
**Hospital stay in days**	14.5 (11–21.8)	16.5 (10.8- 26.8)	15.5 (10.3-22.5)	0.96
**ICU stay in days**	1 (1–3)	2.5 (1.3- 5)	1 (1–3.8)	0.07
**Acute Kidney Injury** ¤	7 (17.5%)	5 (25%)	6 (30%)	0.53
**Death**	2 (5%)	1 (5%)	2 (10%)	0.74

*p compares the 3 treatment groups.

R: randomization. 48 h: 48 hours after randomization. 96 h: 96 hours after randomization. ICU: Intensive care Unit.

¤ In stage 1: n = 15; in stage 2: n = 3 (n = 2 in EPO 40’000 and n = 1 in EPO 20’000 groups).

Incidence of AKI was 22.5% in the studied population (n = 18). Most AKI episodes were stage 1 (n = 15) and stage 2 (n = 3) and occurred during the first week of admission. No AKI episodes required renal replacement therapy. Five patients died early during the follow up period (mortality 6.25%). ICU stay was usually short (few hours to 20 days) and hospital stay ranged from 5 to 45 days. No secondary effect could be assigned to r-HuEPO injection during the follow up and no thrombotic event was recorded.

Hemoglobin, renal parameters and clinical outcome according to EPO treatment are presented in Table 2.

**Figure 2:**
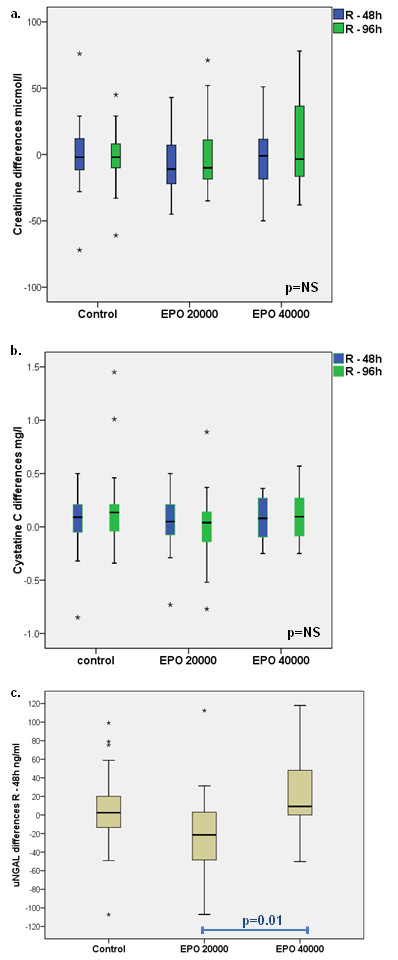
**Changes in renal biomarkers at randomization, 48 h (R-48 h) and 96 h (R-96 h) according to treatment group.** Panels a, b and c represent creatinine, cystatine C and urinary NGAL differences, respectively (*outliers). NS: non significant.

R-HuEPO at either of the two doses had no significant effect on the incidence of AKI. Moreover, r-HuEPO treatment did not significantly modify the renal function parameters when compared to control patients as assessed by the absolute creatinine, cystatin c and urinary NGAL levels at 48 and 96 hours, in each group. There were no significant differences between the groups at any time during follow-up with respect to changes in creatinine, cystatin c and urinary NGAl levels. Although there was a significant difference in the change of urinary NGAL levels between the EPO 20’000 and the EPO 40’000 group (p = 0.01), there was no significant differences with the placebo group (Figure 2).

We pooled the two EPO doses and confirmed that r-HuEPO did not significantly modify uNGAL median levels between 48 hours and randomization: 2.5 ng/ml (−17.30; 22.50) vs 0.7 ng/ml (−31.78; 25.15); p = 0.767. Creatinine and cystatin C levels changed equally in both groups at all time points. Incidence of AKI during follow up using AKIN criteria was similar in placebo and rh-HuEPO treated patients. Cytokines levels were also similar during follow up in both groups. R-HuEPO did not influence outcomes such as death, length of hospital or ICU stays.

Hemoglobin levels, at randomization and at hospital discharge, were the same in the 3 groups.

Patients diagnosed with AKI during follow-up presented with significantly higher creatinine, cystatine C and urinary NGAL levels at ICU admission (randomization time) as shown in Table 3. The levels of all renal biomarkers remained higher during the 48 h-96 h period in patients with AKI.

**Table 3:** Levels of renal biomarkers during follow up classified by diagnosis of AKI

Variables*	**No AKI**	**AKI**	**P**
	**N = 62**	**N = 18**	
**Creatinine R μmol/l**	79.4 ± 19.8	116 ± 39.9	=0.001
**Creatinine 48 h μmol/l**	74.0 ± 19.7	142.1 ± 50.8	<0.001
**Creatinine 96 h μmol/l**	74.1 ± 18.1	129.7 ± 46.4	<0.001
**Cystatin C R mg/l**	1.14 ± 0 .24	1.67 ± 0.44	<0.001
**Cystatin C 48 h mg/l**	1.20 ± 0.27	1.73 ± 0.36	<0.001
**Cystatin C 96 h mg/l**	1.20 ± 0.24	1.90 ± 0.49	<0.001
**uNGAL R ng/ml**	18.9 (0–43.5)	57.6 (26.5-138.8)	<0.001
**uNGAL 48 h ng/ml**	17.4 (5.8-40.9)	40.2 (21.1-102.6)	0.009

R: randomization. 48 h: 48 hours after randomization. 96 h: 96 hours after randomization. AKI: acute kidney injury.

*Cystatine C is expressed as mean ± standard deviation. uNGAL is expressed as median and (25^th^-75^th^ percentiles).

Similarly, the five patients that died during follow-up demonstrated higher levels of both cystatine C (1.76 +/−0.47 mg/l vs. 1.23+/−0.34 mg/l, p = 0.004) and urinary NGAL (103.2 ng/ml vs. 22.2 ng/ml, p = 0.004) at randomization, whereas creatinine levels were not statistically different (86.6 ± 28.8 μmol/l vs. 102.3 ± 33.3 μmol/l, p = 0.24).

### Evolution of plasma IL6 and IL8 in the treatment groups

**Figure 3:**
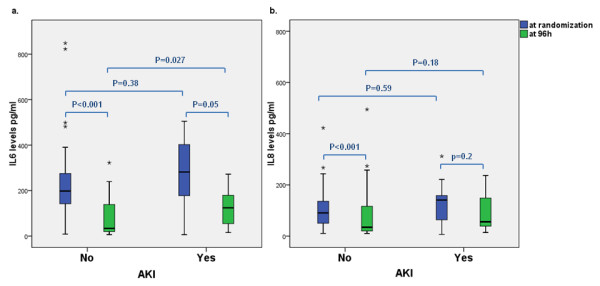
Interleukin levels over time in presence or absence of AKI. Panel a and panel b represent IL6 and IL 8 at randomization and 96 hours, respectively, in patient with and without AKI (*outliers). p compares groups using log transformation.

IL6 and IL8 levels were similar at randomization between placebo and EPO treated patients. We obtained the same results when the three groups were analyzed separately (Table 4). The levels of these cytokines significantly decreased during follow-up in all groups. No difference was observed on the levels of these cytokines at either time-point after r-HuEPO treatments. Furthermore, IL6 and IL8 levels at randomization were similar in all patients regardless of AKI (Figure 3), whereas IL6 levels at 96 hours were significantly higher in patients with AKI [124.5 (51.7-213.9)pgl/ml vs 33.8 (19.8-138.9)pg/ml, p = 0.027] (Figure 3) IL6 levels at randomization were much higher in patients who died [947 (351.7-1311.1) vs 198 (142.7-283.2), p = 0.007], while IL8 levels were slightly higher [197.1 (159.9-189.1) vs 87.9 (49.8-137.5), p = 0.046].

**Table 4:** Levels of interleukins during follow-up

Variables *	**All**	**Control**	**EPO 20’000**	**EPO 40’000**	**P∥**
**pg/ml**	**N = 80**	**N = 40**	**N = 20**	**N = 20**	
**IL-6 R**	207.7 (144.1-301.2)	177.8 (104.7-351.4)	229.5 (159.8-342.9)	250 (177.8-280.3)	0.54
**IL-6 96 h**	51.7 (20.5-143.4)	48.3 (18.9-139.9)	129.6 (20.5-248.6)	50.7 (27.7-135.3)	0.46
**IL-8 R**	98.4 (50.4-154.3)	98.4 (46.3-142.8)	105.4 (56–220.4)	96.6 (52.0-148.9)	0.81
**IL-8 96 h**	40.1 (21.9-124.4)	41.4 (22,5-121.1)	62.3 (20.9-172.4)	30.9 (17.7-104.7)	0.40

*Expressed as median (25^th^-75^th^ percentiles).

R: randomization. 96 h: 96 hours after randomization.

**∥** p compares the 3 treatment groups using log transformation.

## Discussion

R-HuEPO treatment has renoprotective properties in experimental ischemic renal injury when given before, during or even after the injury [5-7]. The effect of r-HuEPO on prevention and treatment of AKI in patients is still very controversial. In our study, we gave r-HuEPO shortly after cardiac surgery which was inefficient in preventing mild forms of AKI, independent of the dose used. Moreover, EPO did not modify NGAL urinary levels, nor did it reduce hospital stay and mortality. This contrasts with a recent study [23] where erythropoietin given at the time of operation was efficient in preventing acute kidney injury, reducing ICU stays and decreasing mortality in cardiac surgery patients. However, timing of administration and type of r-HuEPO (epoetin-beta) differed from our study protocol. Another interventional study in high risk ICU patients, including patients with miscellaneous causes of kidney injuries, demonstrated that EPO was ineffective at preventing mild or severe AKI and did not modify mortality and need for dialysis [24], similarly to our study. Notably in this study, patients were selected based on the rise in tubular biomarkers, and therefore were probably also treated after the onset of renal injury. In kidney transplant recipients, EPO administered prior to transplantation did not decrease the rate of delayed graft function [25]. Altogether, these studies show that erythropoietin may have some beneficial effect in human AKI when given before the ischemic injury. However, even an early post injury treatment, in opposition to what is observed in rodents, appears ineffective. This would limit the applicability of EPO treatment post-intervention in prevention of AKI in ICU patients or should lead to a more thorough identification of high-risk patients before intervention or before other procedures with a renal risk such as contrast media administration. Nevertheless, these conclusions are supported only by one positive monocentric study [23] and more data are necessary. The ongoing EPO-TBI study (NCT 00987454) may provide an answer to this question. This study aims to include 606 patients having traumatic brain injury to evaluate EPO for brain protection but renal biomarkers will be also analyzed [8]. Finally, a study testing the effect of r-HuEPO using a scoring system identifying high-risk patients would be desirable.

Change in urinary NGAL levels was a primary outcome and pooled data showed no effect of r-HuEPO on uNGAL levels compared to placebo during the observation time. We observed that α-Epoetin 20’000ui had a tendency toward decreasing urinary NGAL in comparison to the higher dose, but not in comparison to placebo. The difference between high and low doses of r-HuEPO has not been described previously and we do not have a clear explanation for this result. This could be a statistical difference due to the small sample as clinically relevant parameters (death, length of stay, ICU admission, AKI incidence) were similar between the groups, although we cannot formally exclude a dose-dependent effect. The choice of NGAL levels as a primary outcome was made on the presumption of a better sensitivity of this test for AKI detection. However, establishing the sensitivity of NGAL is difficult given the lack of current gold standard for AKI diagnosis and we believe that NGAL levels over time may be of interest given its prognostic value for outcome [26]. In our patients we only measured NGAL, IL6, IL8 and cystatine C at ICU admission. Baseline values were not available. However, in patients suffering from AKI, both NGAL and cystatine C levels were significantly higher at admission than in non-AKI patients. Similar observations were made in patients that died during the observation period. Despite the low number of events, this tends to confirm that NGAL and cystatine C levels at admission (3–4 hours after surgery) are more valuable than creatinine levels in predicting AKI and mortality, as shown in other studies [13,14,27].

In our study, the incidence of AKI (all stages) was 22.5%, but only three patients suffered from stage 2 AKI and no patient required dialysis. The incidence of severe AKI was low despite the high prevalence of diabetes and CKD and that most of the patients underwent valvular replacement. This may be related to the inclusion of elective patients only due to strict exclusion criteria. Furthermore no nephrotoxic drug was prescribed nor was a contrast agent administered during the observation period that could have increased the rate of renal events. Although 7 patients received contrast medium before surgery they did not have a higher rate of AKI. Recent observations also establish a lower incidence of severe AKI after cardiac surgery than previously reported, despite a remaining important repercussion of low grade AKI on mortality and costs [28]. This may be due to improvement in anesthetic care and surgical techniques. Given this limitation, we cannot demonstrate a protective effect of r-HuEPO on severe AKI after cardiac surgery. However, our study demonstrates that stage 1 AKI, a relatively frequent condition after heart surgery that is correlated to mortality, length of stay and costs, is not prevented by r-HuEPO treatment.

Erythropoietin has been shown to have immunomodulatory effects [19,20,29]. In this study, IL6 and IL8 levels were elevated postoperatively and decreased thereafter. Administration of r-HuEPO shortly after surgery did not modify the plasma profile of these cytokines. As IL6 is emerging as a predictive factor for acute kidney injury and adverse post-operative outcomes [17,30], the absence of a beneficial effect of EPO on its levels parallels the unchanged incidence of acute kidney injury and mortality. This, together with the absence of a protective effect on renal function, does not support the use of r-HuEPO to decrease the anti-inflammatory response.

Our study has several limitations. First the number of events was quite small and for that reason the study may be underpowered even with the use of a sensitive primary outcome measure of change in urinary NGAL levels since the standard deviation of uNGAl was higher than expected. Secondly, we cannot exclude the presence of elevated NGAL levels before randomization, especially in patients with chronic kidney disease or diabetes, as blood was not taken from patients prior to surgery. Nevertheless diabetic patients did not show increased levels of uNGAL at randomization. As for CKD patients, data are difficult to analyze because of a small sample size. In each treatment group, the uNGAL level was higher at randomization in patients with CKD but the change in uNGAL level at 48 h and randomization or between treatment groups was not significantly different. Finally, we did not correct uNGAL for urinary creatinine levels, as this correction did not modify sensitivity of uNGAL in similar settings [27]. Nevertheless, this study is one of the few randomized studies studying EPO effects on renal function and is the only one testing the effect of EPO on inflammatory markers such as cytokines.

## Conclusion

In conclusion, administration of two different doses of α-Epoetin:to adult patients after cardiac surgery did neither decrease markers of renal injury or renal function such as urinary NGAL, cystatine C and creatinine, nor modify the incidence of stages 1 and 2 AKI. In addition, EPO did not modify pro-inflammatory cytokine levels. Erythropoietin administered after an ischemic injury is probably ineffective in patients to prevent low stage AKI and improve survival.

## Abbreviations

EPO: Erythropoeitin; r-HuEPO: Recombinant human erythropoietin; AKI: Acute kidney injury; NGAL: Neutrophil gelatinase-associated lipocalin; uNGAL: Urinary Neutrophil gelatinase-associated lipocalin; CKD: Chronic kidney disease; ICU: Intensive care unit.

## Competing interests

The authors declare that they have no competing interest.

## Authors’ contributions

SdS and BP collected the data, analyzed the results and wrote the manuscript. LW collected the data. JP and JAR contributed to designing the study and recruiting the patients. PYM and PS designed the study, collected the data and revised the manuscript. All authors read and approved the final manuscript.

## Pre-publication history

The pre-publication history for this paper can be accessed here:

http://www.biomedcentral.com/1471-2369/13/132/prepub

## References

[B1] Arcasoy MO The non-haematopoietic biological effects of erythropoietin Br J Haematol 2008 141 1 14 31 10.1111/j.1365-2141.2008.07014.x 18324962

[B2] Brines M The therapeutic potential of erythropoiesis-stimulating agents for tissue protection: a tale of two receptors Blood Purif 2010 29 2 86 92 10.1159/000245630 20093809

[B3] Johnson DW Pat B Vesey DA Guan Z Endre Z Gobe GC Delayed administration of darbepoetin or erythropoietin protects against ischemic acute renal injury and failure Kidney Int 2006 69 10 1806 1813 10.1038/sj.ki.5000356 16598197

[B4] Uchino S Kellum JA Bellomo R Acute renal failure in critically ill patients: a multinational, multicenter study JAMA 2005 294 7 813 818 10.1001/jama.294.7.813 16106006

[B5] Vesey DA Cheung C Pat B Endre Z Gobe G Johnson DW Erythropoietin protects against ischaemic acute renal injury Nephrol Dial Transplant 2004 19 2 348 355 10.1093/ndt/gfg547 14736958

[B6] Sharples EJ Patel N Brown P Erythropoietin protects the kidney against the injury and dysfunction caused by ischemia-reperfusion J Am Soc Nephrol 2004 15 8 2115 2124 10.1097/01.ASN.0000135059.67385.5D 15284297

[B7] Yang CW Li C Jung JY Preconditioning with erythropoietin protects against subsequent ischemia-reperfusion injury in rat kidney FASEB J 2003 17 12 1754 1755 12958199 10.1096/fj.02-1191fje

[B8] Moore E Bellomo R Erythropoietin (EPO) in acute kidney injury Ann Intensive Care 2011 1 1 3 10.1186/2110-5820-1-3 21906325 PMC3159901

[B9] Mehta RL Kellum JA Shah SV Acute Kidney Injury Network: report of an initiative to improve outcomes in acute kidney injury Crit Care 2007 11 2 R31 10.1186/cc5713 17331245 PMC2206446

[B10] Ricci Z Cruz D Ronco C The RIFLE classification for acute kidney injury definition Am J Surg 2009 198 1 152 153 10.1016/j.amjsurg.2008.06.033 18805517

[B11] Mariscalco G Lorusso R Dominici C Renzulli A Sala A Acute kidney injury: a relevant complication after cardiac surgery Ann Thorac Surg 2011 92 4 1539 1547 10.1016/j.athoracsur.2011.04.123 21872837

[B12] Lassnigg A Schmidlin D Mouhieddine M Minimal changes of serum creatinine predict prognosis in patients after cardiothoracic surgery: a prospective cohort study J Am Soc Nephrol 2004 15 6 1597 1605 10.1097/01.ASN.0000130340.93930.DD 15153571

[B13] Haase M Bellomo R Devarajan P Novel biomarkers early predict the severity of acute kidney injury after cardiac surgery in adults Ann Thorac Surg 2009 88 1 124 130 10.1016/j.athoracsur.2009.04.023 19559209

[B14] Haase M Devarajan P Haase-Fielitz A The outcome of neutrophil gelatinase-associated lipocalin-positive subclinical acute kidney injury: a multicenter pooled analysis of prospective studies J Am Coll Cardiol 2011 57 17 1752 1761 10.1016/j.jacc.2010.11.051 21511111 PMC4866647

[B15] Koyner JL Vaidya VS Bennett MR Urinary biomarkers in the clinical prognosis and early detection of acute kidney injury Clin J Am Soc Nephrol 2010 5 12 2154 2165 10.2215/CJN.00740110 20798258 PMC2994075

[B16] Park M Coca SG Nigwekar SU Garg AX Garwood S Parikh CR Prevention and treatment of acute kidney injury in patients undergoing cardiac surgery: a systematic review Am J Nephrol 2010 31 5 408 418 10.1159/000296277 20375494 PMC2883845

[B17] Chawla LS Seneff MG Nelson DR Elevated plasma concentrations of IL-6 and elevated APACHE II score predict acute kidney injury in patients with severe sepsis Clin J Am Soc Nephrol 2007 2 1 22 30 17699383 10.2215/CJN.02510706

[B18] Gueret G Lion F Guriec N Acute renal dysfunction after cardiac surgery with cardiopulmonary bypass is associated with plasmatic IL6 increase Cytokine 2009 45 2 92 98 10.1016/j.cyto.2008.11.001 19128984

[B19] Bian XX Yuan XS Qi CP Effect of recombinant human erythropoietin on serum S100B protein and interleukin-6 levels after traumatic brain injury in the rat Neurol Med Chir (Tokyo) 2010 50 5 361 366 10.2176/nmc.50.361 20505289

[B20] Shen Y Wang Y Li D Recombinant human erythropoietin pretreatment attenuates heart ischemia-reperfusion injury in rats by suppressing the systemic inflammatory response Transplant Proc 2010 42 5 1595 1597 10.1016/j.transproceed.2009.11.050 20620481

[B21] Licker M Diaper J Cartier V Clinical Review: Management of weaning from cardiopulmonary bypass after cardiac surgery Ann Card Anaesth 2012 15 3 206 223 10.4103/0971-9784.97977 22772515

[B22] Pedersen KR Ravn HB Hjortdal VE Norregaard R Povlsen JV Neutrophil gelatinase-associated lipocalin (NGAL): validation of commercially available ELISA Scand J Clin Lab Invest 2010 70 5 374 382 10.3109/00365513.2010.486868 20509756

[B23] Song YR Lee T You SJ Prevention of acute kidney injury by erythropoietin in patients undergoing coronary artery bypass grafting: a pilot study Am J Nephrol 2009 30 3 253 260 10.1159/000223229 19494484

[B24] Endre ZH Walker RJ Pickering JW Early intervention with erythropoietin does not affect the outcome of acute kidney injury (the EARLYARF trial) Kidney Int 2010 77 11 1020 1030 10.1038/ki.2010.25 20164823

[B25] Martinez F Kamar N Pallet N High dose epoetin beta in the first weeks following renal transplantation and delayed graft function: Results of the Neo-PDGF Study Am J Transplant 2010 10 7 1695 1700 10.1111/j.1600-6143.2010.03142.x 20642691

[B26] Hall IE Coca SG Perazella MA Risk of Poor Outcomes with Novel and Traditional Biomarkers at Clinical AKI Diagnosis Clin J Am Soc Nephrol 2011 6 12 2740 2749 10.2215/CJN.04960511 22034509 PMC3255362

[B27] Mishra J Dent C Tarabishi R Neutrophil gelatinase-associated lipocalin (NGAL) as a biomarker for acute renal injury after cardiac surgery Lancet 2005 365 9466 1231 1238 10.1016/S0140-6736(05)74811-X 15811456

[B28] Dasta JF Kane-Gill SL Durtschi AJ Pathak DS Kellum JA Costs and outcomes of acute kidney injury (AKI) following cardiac surgery Nephrol Dial Transplant 2008 23 6 1970 1974 10.1093/ndt/gfm908 18178605

[B29] Solling C Christensen AT Krag S Erythropoietin administration is associated with short-term improvement in glomerular filtration rate after ischemia-reperfusion injury Acta Anaesthesiol Scand 2011 55 2 185 195 10.1111/j.1399-6576.2010.02369.x 21226860

[B30] Liu KD Altmann C Smits G Serum interleukin-6 and interleukin-8 are early biomarkers of acute kidney injury and predict prolonged mechanical ventilation in children undergoing cardiac surgery: a case–control study Crit Care 2009 13 4 R104 10.1186/cc7940 19570208 PMC2750143

